# What influences public views on forensic DNA testing in the criminal field? A scoping review of quantitative evidence

**DOI:** 10.1186/s40246-019-0207-5

**Published:** 2019-05-23

**Authors:** Helena Machado, Susana Silva

**Affiliations:** 1 Institute for Social Sciences, University of Minho, Braga, Portugal; 2 EPIUnit - Instituto de Saúde Pública, Universidade do Porto, Porto, Portugal

**Keywords:** DNA profiling, DNA databases, DNA fingerprinting, Forensic genetics, Public opinion

## Abstract

**Background:**

Forensic DNA testing is a powerful tool used to identify, convict, and exonerate individuals charged of criminal offenses, but there are different views on its benefits and risks. Knowledge about public views on forensic DNA testing applied in the criminal field is socially valuable to practitioners and policymakers. This paper aims to synthesize quantitative evidence about the factors that influence public views on forensic DNA testing in the criminal field. Based on a systematic search conducted in January 2019, a scoping review was performed, targeting studies presenting original empirical data that were indexed in Web of Science and PubMed. The two authors performed eligibility and data extraction.

**Results:**

The 11 studies were conducted mainly in European countries (Italy, Portugal, Serbia, Spain, Switzerland) and the remaining derived from the USA and New Zealand. Non-representative samples were mostly used to explore the benefits and risks of criminal DNA databases, criteria for insertion and retention of DNA samples and profiles, knowledge, willingness to donate a DNA sample, and custody. The value of forensic DNA databases in protecting society from crime was emphasized. Concerns about improper access to forensic genetic data and risks to civil liberties associated with its uses were expressed. The scarce literature on Forensic DNA Phenotyping and familial searching revealed the same trend of positively valuing forensic DNA testing. Only factors related with socioeconomic position were assessed by more than two studies. Results suggested that public views on forensic DNA testing are influenced by the level of education, age, and exposure to law enforcement occupations although not in a straightforward manner.

**Conclusion:**

Further empirical research should assess standardized factors related with social and structural levels (e.g., scientific literacy, public trust in the justice system and concerns about victimization or police activity) and be performed in different national jurisdictions to enable generalization and comparison of findings. It is needed to expand empirical studies on public views about the commercialization of forensic science and the use of recent controversial techniques and new transparency and accountability models.

## Background

Forensic DNA testing has become a significant resource for criminal investigation and prosecution activities in criminal justice systems throughout the world [1–4]. Forensic DNA testing can be conducted in several ways: first, by comparing the DNA profiles from criminal suspects’ to DNA evidence, so as to assess the likelihood of their involvement in a crime. The second kind of use is related to searching for a link between the biological material collected from a crime scene to a DNA profile stored in a criminal DNA database. The third form of forensic DNA testing is related to procedures to search for criminal suspects through their connection with biological relatives. Finally, the inference of human externally visible physical features from a biological sample collected at the crime scene [5, 6].

One prominent aspect of forensic DNA testing is the establishment and expansion of centralized national criminal DNA databases. These databases involve the collection, storage, and use of DNA profiles from nominated suspects, convicted offenders, victims, volunteers, and other persons of interest to criminal investigation work. The primary function of a criminal DNA database is to produce matches between individual profiles and crime scene stains, which requires a constant input of both. Around 69 countries currently operate national forensic DNA databases, and others are being expanded or established in at least 34 additional countries [7]. With increasing proportions of the population included in forensic DNA databases, several authors suggested that more research should be aimed at finding ways to evaluate and monitor their performance [8–10] including the assessment of public views [11–13].

Recent innovations and developments in forensic DNA testing in the criminal field are related to techniques of Forensic DNA Phenotyping (FDP), the use of ancestry-informative markers, and familial searching. FDP can be described as a set of techniques that aims to infer human externally visible physical features—eye, hair, and skin color—and continental-based biogeographical ancestry of criminal suspects on the basis of analysis of biological materials collected at crime scenes [14, 15]. FDP techniques have been applied in various jurisdictions in a limited number of high-profile cases to provide intelligence for criminal investigation [16, 17]. Familial searching makes use of procedures to detect genetic relatedness in criminal DNA databases to search for criminal suspects through their connection with biological relatives [18, 19].

Different views on the capabilities, benefits, and risks of forensic DNA testing circulate within modern societies. Supporters of the expansion of forensic DNA testing in the criminal justice system invoke its capacity to serve as a valuable law enforcement tool, namely by improving efficiency in fighting crime, helping in the prevention of miscarriages of justice and deterrence of criminal activity, which is, in turn, expected to reduce crime and increase public safety and security [20–22]. Critics concerned with potential threats to civil liberties argue that forensic DNA testing, in particular the storage of profiles in computerized databases operating as forensic DNA databases for criminal identification, may threaten the protection of a range of human rights, in particular liberty, autonomy, privacy, informed consent, moral and physical integrity, and the presumption of innocence [5, 12, 23, 24].

Other risks reported in the literature in regards to forensic DNA testing are the following: social stigmatization and racial stereotyping due to the overrepresentation of specific social and ethnic groups in the criminal DNA databases [25, 26]; concerns that data processing may be associated with individual or group characteristics or criminal behavior, and therefore lead to discrimination [27]; and mistaken identification and wrongful conviction resulting from erroneous interpretations of the information provided by DNA profiles [2, 28, 29]. There are also several problematic issues related to the transnational exchange of DNA data in the context of police and judiciary cooperation. Some issues relate to lack of transparency on the uses of DNA data, risk of false positives, lack of standardization on DNA analysis among different countries, lack of ethical oversight of the transnational flow of law enforcement information, and potential violations of data protection regulations [6, 30–32]. Finally, the presentation of DNA evidence in courts before the assumed deficit of knowledge from the part of non-experts, along with over-expectations towards the capability of DNA evidence to solve criminal cases, is also considered a critical aspect of the presence of forensic genetic testing in the criminal justice system [6, 30–32]. Literature in the field of forensic sciences has consistently reported the challenges of communicating probabilistic results and likelihood ratios related to DNA evidence in typical identification casework to the court [33–35].

Ethical concerns also apply to Forensic DNA Phenotyping (FDP), namely the potential to increase risks of stigmatization and reinforcement of the criminalization of specific populations more vulnerable to the action of the criminal justice system, and the sensitive nature of disclosing information related to physical characteristics of potential suspects [36–38]. Besides, ethical controversies related to FDP apply to the selection of criminal cases that justify its application and the need to develop reflections about the implications of developments of FDP in the realm of the criminal justice system [16, 17].

Familial searching raises ethical, technical, logistical, and efficacy questions. One central issue is linked to the economic, temporal, and human resources needed to search, review, and refine the selection and monitoring of the pool of hundreds of potential suspects [39, 40]. Ethical objections to familial search methods tend to be raised on the grounds of privacy since familial searches constitute an expansion of the net of genetic surveillance to persons whose genetic information would have remained private from the State had it not been for the actions of their blood relatives [41, 42]. Scrutiny and assessment of the costs and benefits of familial search method lack, in particular in a context where law and policy oversight of familial searches in recreational genealogy databases has been neglected [43–45].

Knowledge about existing research on public views on forensic DNA testing is thus essential to inform ethically sustainable governance models [12]. Understanding public views is also socially valuable to practitioners, policymakers, and other professional categories who represent what Williams and Wienroth [13] have interpreted as a different type of “informed” public, i.e., those who do not necessarily have technical familiarity with DNA technologies, but have several motives of interest towards these technologies. The establishment of national DNA databases and the development of practices of forensic DNA testing are typically launched without any prior consultation with the public [11, 13, 46, 47].

The only existing review on public views on forensic DNA testing in the criminal field is authored by Amankwaa [46]. This work mainly focused on general trends related to the perception of the criteria for inclusion of profiles and the periods of time and conditions for their retention and/or deletion and the underlying reasons. It did not explore quantitative findings about the factors that influence public perspectives. Additionally, this published review did not cover the themes of forensic phenotyping and familial searching. This paper fills this gap, by synthesizing quantitative evidence about the factors that influence public views on forensic DNA testing in the criminal field.

## Methods

We followed the guidance for descriptive reviews by Levac et al. [48], based on the methodological framework developed by Arksey and O’Malley [49].

### Stage 1: Identifying the research question

The central question guiding this scoping review is: What are the main factors influencing public views on forensic DNA testing in the criminal field?

### Stage 2: Identifying relevant studies

A search of the publications on two electronic databases (PubMed® and Web of Science™) was conducted in January 2019, with no restriction set for language or time of publication, using the following search expression: (“DNA database” OR “DNA databases” OR “genetic genealogy databases” OR “DNA profiling” OR “DNA fingerprinting” OR “familial searching” OR “forensic DNA phenotyping”) AND (“public opinion” OR “public attitudes” OR “public perception” OR “public understanding” OR “public perspectives” OR “public views” OR “survey”). The search was followed by backward reference tracking, examining the references of the selected publications based on full-text assessment.

### Stage 3: Study selection

The inclusion criteria allowed only empirical, peer-reviewed, full-length, original quantitative studies reporting data on the factors influencing public views on forensic DNA testing. The titles of 452 records were retrieved. After the removal of the duplicates, 363 records were examined. The two authors independently screened all the papers retrieved initially, based on the title and abstract and afterward, based on full-text. This was crosschecked and discussed in both phases, and a perfect agreement was achieved.

**Fig. 1:**
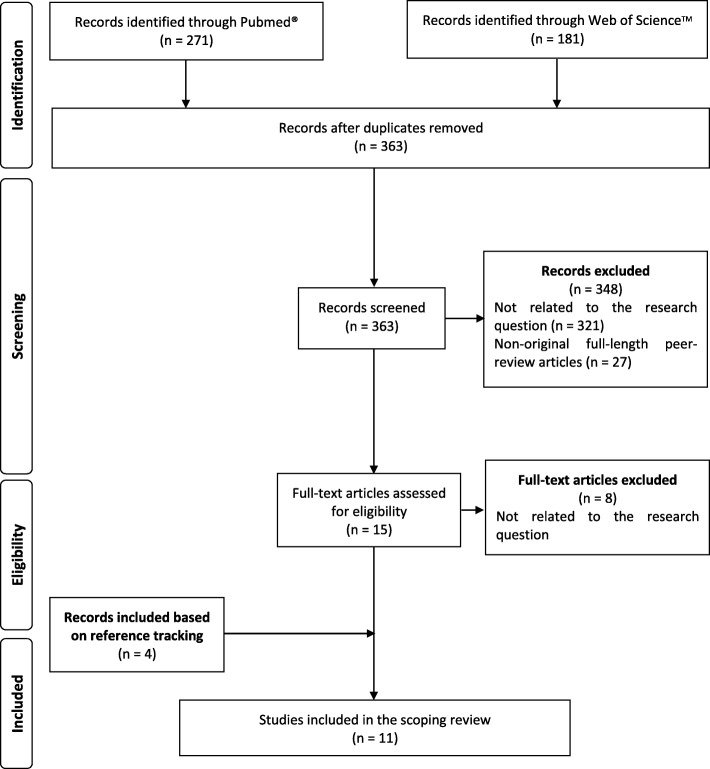
Flowchart showing the search results and screening process

The screening process is summarized in Fig. 1. Based on the title and abstract assessments, 348 records were excluded, because they were neither original full-length peer-review empirical studies nor explored quantitative data about variables influencing the public views on forensic DNA testing in the criminal field. Of the 15 fully read papers, 7 met the inclusion criteria. After the backward reference tracking, 4 papers were included, and the final review was composed of 11 papers.

### Stage 4: Charting the data

A standardized data extraction sheet was developed and completed by the authors. Descriptive data for the characterization of studies included information about the authors and publication year, the country where the study was developed, study aim, participants and sample, and methods for data collection. We also retrieved quantitative data on variables whose association with the public views on forensic DNA testing in the criminal field was tested and reported. Only the directions of the statistically significant associations were registered.

### Stage 5: Collating, summarizing and reporting the results

The main characteristics of the 11 studies included can be found in Table 1, ordered by year of publication. The quantitative findings regarding the factors whose influence on the public views on forensic DNA testing in the criminal field was tested are presented in Table 2.

**Table 1:** Main characteristics of the quantitative studies examining the factors influencing the public views on forensic DNA testing in the criminal field (*n* = 11)

Authors, year of publication	Country	Aim	Sample	Methods for data collection	Variables assessed
Dundes, 2001 [50]	The USA	To assess whether the American public would support the collection of DNA samples from various segments of the population (from convicted violent offenders to all newborns)	Convenience sample 416 persons living in Maryland, aged 18 years or older	Questionnaire	Gender, age, level of education, race, living area (urban, suburban, rural), ever been frisked, attitude towards capital punishment, confidence in DNA technology, willingness to resort to any means necessary to curb crime
Gamero et al., 2007 [51]	Spain	To analyze the opinion of the Spanish population with regard to the circumstances that would justify the inclusion of biological samples and DNA analyses of individuals in a genetic database	Representative sample 1654 participants, from 15 years of age upwards	Questionnaire	Gender, age, level of education, occupation ^a^
Gamero et al., 2008 [52]	Spain	To analyze the opinion of the Spanish population with regard to the institutions that should exercise custody and protection over the DNA profile databases	Representative sample 1654 participants, from 15 years of age upwards	Questionnaire	Gender, age, level of education, occupation^a^
Curtis, 2009 [53]	New Zealand	To discuss the expectations and level of knowledge of the New Zealand public of the DNA database	Random sample (telephone directories) 100 participants, aged 16 years or older	Questionnaire	Gender, age, level of education, household income, ethnicity
Curtis, 2014 [54]	New Zealand	To explore public understanding of the forensic use of DNA: sources of knowledge, understandings of processes, and attitudes towards DNA use	Random sample (telephone directories) 394 New Zealand residents, aged 16 years or older	Computer-assisted telephone (landline) questionnaire (closed and open questions)	Gender, age, level of education, household income, ethnicity, political preferences
Machado and Silva, 2014 [11]	Portugal	To analyze the citizens’ willingness to donate voluntarily a sample for profiling and inclusion in the National Forensic DNA Database and the views underpinning such a decision	Judgment sample 628 participants, aged between 17 and 82 years	Online questionnaire (closed and open questions)	Gender, age, level of education, occupation^b^
Machado and Silva, 2015 [55]	Portugal	To assess the influence of the professional group, education, and age on public perspectives on the risks and benefits of forensic DNA databases	Judgment sample 628 participants, aged between 17 and 82 years	Online questionnaire (closed and open questions)	Age, level of education, occupation^c^
Zieger and Utz, 2015 [56]	Switzerland	To draw a broader picture of the public opinion on DNA databasing and to contribute to the debate about the possible future uses of genetics to reveal phenotypic characteristics	Convenience and snowball sample 284 German-speaking Swiss residents, aged between 18 to 72 years	Online questionnaire (closed and open questions)	Gender, age, level of education, nationality, occupation^d^
Teodorovic et al., 2017 [47]	Serbia	To instigate a consultation with the Serbian public regarding their views on various aspects of the forensic DNA databank (custody, DNA sample and profile inclusion and retention criteria, ethical issues and concerns)	Convenience and stratified sample 558 participants, aged between 19 and 65 years	Questionnaire	Gender, age, level of education, occupation^e^
Tozzo et al., 2017 [57]	Italy	To assess knowledge about biobanks, perception of the related benefits and risks, willingness to donate samples to a biobank for research purposes, attitude to having DNA profile included in a forensic DNA database and the underlying reasons	Homogeneous sample 959 students from Padua University, aged between 19 and 24 years	Questionnaire	Gender, type of university course (law, medicine, professional nursing)
Guerrini et al., 2018 [43]	The USA	To assess public opinion on police access to genetic genealogy websites and customer information from DTC genetic testing companies	Crowdsourcing recruitment^f^ 1587 participants, aged between 18 and 88 years	Online questionnaire	Gender; age; race/ethnicity; household income; use of genealogy websites to research relatives; purchase of DTC genetic testing services; personal or relative’s victimization, arrest, or criminal conviction; personal or relative’s employment in law enforcement

*DTC* direct-to-consumer

^a^Professionals working in the fields of law, health, security, and a group representing all other professions

^b^Professionals working in the fields of law enforcement, health and life sciences, research and development, other professions (professional group was excluded from the analysis because 31.8% of the participants did not report that information)

^c^Professionals working in the fields of law enforcement, health and life sciences, research and development, other professions

^d^Working or not working in the field of police, judicature or forensics

^e^Staff of prosecutors’ offices, prisoners, prison guards, police officer students, general public (a population subgroup without any known prior professional association with forensic DNA databases)

^f^Participants were recruited using the online marketplace Amazon Mechanical Turk (MTurk)

**Table 2:** Main findings of quantitative studies on the factors influencing the public views of forensic DNA testing in the criminal field (*n* = 11)

Factors	Topics					
	Benefits, risks, and ethical concerns	Criteria for insertion and retention of DNA profiles	Knowledge	Willingness to voluntarily donate a DNA sample	Custody and control	DNA phenotyping and familial searching
Level of education	• More educated: less likely to agree that forensic DNA database can influence in developing swifter and more accurate justice [55] • More educated: stronger confidence in the impact of a DNA database in crime fighting [47]	• More educated: more likely to support DNA databases for convicted violent offenders [50] • More educated: more likely to not accept a universal database covering all Swiss residents [56]	• More educated: increase awareness of the use of DNA profiling in the identification of persons [52]	• More educated: less willing to donate [11] • More educated: higher unwilling to donate [56]	• More educated: less support for Local and State Security Agencies as custodians of the databases [52]	
Age	• Older: increased concerns over the risk of possible uses of the genetic material for purposes other than criminal investigation [55] • Youngest: more likely to select stigmatization and discrimination as risks, while devaluing lack of security and control [55] • Older: more optimistic about the importance of a forensic DNA repository [47]		• Older: more likely to know that the National DNA database exist [56]	• Older: less willing to donate [11]	• Oldest (> 65) and youngest (15–24 years): more support for Local and State Security Agencies [52] • Older: more favored entrusting the national DNA database to an independent entity [47]	
Occupation	• Staff of prosecutor’s offices (vs. general public and prisoners): placed significantly more value on DNA database as a crime-fighting tool; agreed more that a DNA database would not intrude on individuals’ privacy [47]	• Legal professionals (vs. health and local/national security professionals): less support for a Spanish DNA databank for all citizens without their consent; specific groups of non-consenting individuals who repeat the same offense, of whatever the nature or gravity; recidivist offenders found guilty of committing crimes against the lives, integrity, and safety of citizens [51] • Staff of prosecutor’s offices (vs. general public): preferred indefinite storing of convicted offenders’ DNA profiles [47]	• Work in the field of police, law, and forensics: more frequently knew about the existence of the Swiss DNA database [56]	• Law students (vs. students of medicine or professional nursing): less willing to donate to a research biobank [57]		
Gender		• Women: more likely to accept a universal database [56]		• Women: more willing to donate [56]- Women: more willing to donate to a research biobank [57]		• Women: more likely to support police access to genetic genealogy databases [43]
Ethnicity/race	• European descents: more likely to strongly agree that the use of DNA is a great step forward [53] • European descents (vs. Mãori and Pacific): exhibit greater trust and less concerns about ethical and privacy issues (completely trust the owners of the DNA database, would be happy to give a sample if requested by the police, have no concerns about the use of DNA for another purpose or ethical issues around DNA use or eventual occurrence of mistakes) [54]	• Whites (vs. Blacks): more likely to strongly support DNA databases for convicted violent offenders [50]European descents: more likely to be interested in having DNA stored, in particular the DNA of offenders convicted of violent crime [53]	• European descents: more likely to cite newspapers as source of information [53] • European descents (vs. Mãori and Pacific): more likely to identify the sources from which forensic DNA samples are taken, know that DNA is stored, have gained knowledge from newspapers [57] • Pacific people were less likely to hear about DNA use [54]			
Political orientation	• Conservative (republican-like) voters (vs. liberal): more likely to completely trust that a sample taken would be used appropriately, agree that the use of DNA is a great step forward, have no concerns about the use of DNA for another purpose or cultural issues around DNA use [54]					
Attitude towards crime control		• Willingness to resort to any means necessary to curb crime and support for capital punishment: best predictors of support for DNA databases for convicted violent offenders [50]				
Being a prisoner		• Prisoners (vs. general public and prosecutor’s office staff) favored profiles of the entire population or no one is included in the national register, storing DNA profiles of individuals convicted for/suspected of having committed serious crimes only, DNA profile being expunged at the end of the prison sentence [47]				

## Results

### Study characteristics

#### Country of study and year of publication

Most quantitative studies were conducted in European countries—Spain [51, 52], Portugal [11, 55], Switzerland [56], Serbia [47], Italy [57]—and the remaining derived from the USA [43, 50] and New Zealand [53, 54]. The studies were published between 2001 [50] and 2018 [43].

#### Sample

Almost all studies used nonprobability sampling techniques, in particular, convenience samples [47, 50, 56], purposive samples [11, 55], a random sample based on telephone directories [53, 54], and one crowdsourcing recruitment using an online marketplace [43]. The samples varied from 100 participants [53] to 1587 participants [43]. A representative sample composed by 1654 participants was used in the two papers related to the study conducted in Spain [51, 52].

#### Topics for assessment

The topic more frequently addressed was the public views on the benefits and risks of criminal DNA databases [47, 53–57], followed by the public perspectives regarding the following issues: criteria for insertion and retention of DNA samples and profiles in criminal DNA databases [47, 50, 51], level of knowledge about forensic DNA testing [53, 54, 57], and willingness to donate a DNA sample for profiling and inclusion in a national forensic DNA database and the reasons underpinning such views [11, 54, 57]. Two studies explored public opinion about the institution that should be given the responsibility for exercising custody over biological samples and the DNA profiles obtained from these samples and protecting and maintaining data confidentiality [47, 52]. The study by Guerrini et al. [43] assessed perspectives on police access to genetic genealogy websites and customer information from DTC genetic testing companies, while Zieger and Utz [56] explored public opinion about uses of genetics to reveal phenotypic characteristics.

### Factors influencing public views on forensic DNA testing in the criminal field

Only six variables, all related with socioeconomic position, were assessed by more than two studies: gender (*n* = 10) [11, 43, 47, 50–54, 56], age (*n* = 10) [11, 12, 43, 47, 50–54, 56], level of education (*n* = 9) [11, 12, 47, 50–54, 56], exposure to law enforcement occupations or law university courses (*n* = 8) [11, 12, 43, 47, 51, 52, 56, 57], race/ethnicity (*n* = 4) [43, 50, 53, 54], and household income (*n* = 3) [43, 53, 54]. Other factors, in particular those centered on non-professional exposure to the criminal justice system (e.g., personal or relative’s victimization, arrest, or criminal conviction), were assessed once or twice, resulting in inconclusive data.

Those who had more years of education were less willing to voluntarily donate a DNA sample [11, 56] and revealed less support for Local and State Security Agencies as custodians of the databases [52]. They were more aware of the use of DNA profiling in the identification of persons [52] and more likely to support DNA databases for convicted violent offenders [50] but not for all citizens [56]. One study found that more educated participants were less likely to agree that criminal DNA database can influence the development of swifter and more accurate justice [55], but others expressed stronger confidence in the impact of a DNA database in crime fighting [47]. Three studies revealed no association between level of education and public views on forensic DNA testing in the criminal field [51, 53, 54].

Willingness to accept an individual’s own DNA profile insertion decreased markedly with age [11], alongside with more knowledge [56] and increased concern over the risk of possible uses of the genetic material for purposes other than criminal investigation [55] but more optimist perceptions on the importance of a forensic DNA repository [47]. The youngest participants were more likely to select the stigmatization of certain social groups and discrimination in genetic studies as risks while devaluing lack of security and control over access to data contained in the criminal DNA database [55]. One study showed that the older the participants, the more they favored entrusting the national DNA database to an independent entity [47], while Gamero et al. [52] concluded that those with more than 65 years were more likely to support Local and State Security Agencies as custodians of the databases. Five studies revealed no association between age and public views on forensic DNA testing in the criminal field [43, 50, 51, 53, 54].

Studies addressing exposure to law enforcement occupations revealed that those working in the field of police, law, and forensics more frequently knew about the existence of the national DNA database [56]. Legal professionals were less supportive of a universal database without citizen’s consent [51] and tended to perceived DNA database as a crime-fighting tool that would not intrude on individuals’ privacy while preferring indefinite storing of convicted offenders’ DNA profiles [47]. One study found that Law students (vs. students of medicine or professional nursing) were less willing to donate to a research biobank [57]. Four studies revealed no statistically significant association between occupation and the public views on forensic DNA testing in the criminal field [11, 12, 43, 52].

Seven out of the ten studies addressing the influence of gender found no association [11, 47, 50–54]. The remaining three studies revealed consistent results regarding women’s more frequent support to forensic DNA testing, either through the acceptance of a universal database [56] or police access to genetic genealogy websites and customer information from DTC genetic testing companies [43] or by showing more willingness to voluntarily donate a DNA sample [56, 57].

Studies by Curtis reported that persons of European descent were more likely to have knowledge about forensic DNA databases gained from newspapers and tended to exhibit greater trust in the use of DNA and fewer concerns about ethical and privacy issues [53, 54]. They were also more likely to support the storage of DNA of offenders convicted of violent crimes [53], a perspective shared by white participants (vs. black) in the study conducted by Dundes [50]. Guerrini et al. [43] concluded that public opinion on police access to genetic genealogy websites and customer information from DTC genetic testing companies is not influenced by race/ethnicity.

Three additional factors were associated with public views on forensic DNA testing in the criminal field, but all had been reported in only one study: political orientation [54], attitude towards crime control [50], and being a prisoner [47]. Compared to liberal voters, conservative (republican-like) voters were more likely to completely trust that a sample taken would be used appropriately, agree that the use of DNA is a significant step forward, and have no concerns about the use of DNA for another purpose or cultural issues around DNA use [54]. Willingness to resort to any means necessary to curb crime and support for capital punishment were the best predictors of support for DNA databases for convicted violent offenders [50]. Finally, prisoners (vs. general public and prosecutor’s offices staff) favored the following ideas: profiles of the entire population or no-one be included in the national register, storing DNA profiles of individuals convicted for (or suspected of having committed) serious crimes only, and DNA profile being expunged at the end of the prison sentence [47].

## Discussion

This scoping review suggested that quantitative studies about public views on forensic DNA testing in the criminal field explored six main dimensions: (a) benefits, risks and ethical concerns of the uses of forensic DNA testing; (b) criteria for collection of DNA profiles by police forces and circumstances that would justify their insertion and retention in criminal forensic DNA databases; (c) level of knowledge about forensic DNA testing; (d) custody and control of data stored in genetic databases; (e) willingness to voluntarily donate a DNA sample for forensic testing; and (f) circumstances that would justify the use of techniques such as forensic DNA phenotyping and familial searching. Studies tested mainly specific sets of variables related to socioeconomic position and revealed the influence of the level of education, age, and exposure to law enforcement occupations.

The public tends to emphasize the potential benefits of forensic DNA testing in terms of its contribution to fighting crime more efficiently and developing swifter and more accurate justice [46]. This general trend applies to the diverse forms of forensic DNA testing: from conventional techniques (i.e., DNA evidence and forensic DNA databases) to recent innovations in the field (i.e., forensic DNA phenotyping and familial searching). Qualitative studies about public attitudes also showed that forensic DNA testing was seen as the least problematic of genetic applications. Results indicated that while acknowledging human rights issues, the participants tended to prioritize the well-being of society over the risks of a society under excessive surveillance [11, 56, 58–60]. The public’s enthusiasm for forensic DNA testing can be explained by the influence of messages from the media emphasizing the “infallible capacity” of DNA testing to catch criminals [61, 62]. The findings of this scoping review show that while the socioeconomic position has an influence on these general trends, their relationship is not straightforward. The still scarce quantitative evidence about the factors that influence public views on forensic DNA testing thus offers a good opportunity to discuss multiple views on the capabilities, benefits, and risks of these technologies.

Level of education and exposure to law enforcement occupations tended to be more of a predictor of the strength of attitudes towards forensic DNA testing. Previous studies about public perspectives on science and technology, in general, have shown a small but consistent positive correlation between various science literacy measures and support for science and technology, and professional socialization and academic background influence perceptions of the risks of science and technology [63]. However, further research is needed to gain knowledge about the influence of scientific literacy, professional socialization, and academic background in the specific case of public views on forensic science and DNA technologies.

The concrete knowledge about a specific area as a predictor of the strength of attitudes might explain why the professional groups who might have more direct knowledge about the forensic DNA testing—participants working in the field of healthcare and life sciences and professionals in law enforcement and prisoners—are the ones who have stronger views about the benefits and the risks of forensic DNA testing. The lower levels of agreement among law enforcement professionals regarding the capability of forensic DNA testing to contribute towards efficiency in crime fighting and accuracy in the criminal justice system is in line with qualitative studies which demonstrate an enhanced sensitivity among law enforcement professionals regarding the contingencies of forensic work [32, 64]. Other studies revealed that law enforcement professionals and prisoners have a more optimistic view of forensic DNA testing (i.e., emphasizing the benefits). This result also concurs with the results obtained in qualitative studies conducted with professionals from the field of forensic genetics that also showed that stakeholders who work in the criminal justice system and in forensic genetics tend to highlight forensic uses of DNA as highly beneficial resources for fighting crime and improving justice, whereas the ethical risks are relatively devalued [13, 65, 66].

This review highlighted that populations criminalized by the justice system, as well as ethnic minorities, show high levels of awareness of the potential risks of uses of forensic DNA testing, such as fears of social discrimination, excessive state surveillance, and misuse of data. This result follows the findings of previous qualitative studies with ethnic minorities and prisoners [67, 68]. This review revealed also an optimistic view of prisoners in regard to forensic DNA testing, which is in accordance with qualitative studies that showed the high support of groups criminalized by the justice system for the expansion of forensic DNA databases, while expressing that view that forensic DNA testing as powerful tools provided protection against wrongful accusations [69–71].

Regarding the influence of age, some studies included in this review revealed no association between age and the public views on forensic DNA testing in the criminal field, while other studies showed an impact on perceptions of risks. It is noteworthy that the youngest participants showed more concern about the risks of stigmatization of certain social groups and discrimination in genetic studies. This finding is similar to the results obtained by Stackhouse et al. [71] in which it was observed that younger people are more concerned about discrimination and the ethnic bias produced by national forensic DNA databases and less worried about access to, and use of, the genetic information they contain for purposes other than criminal investigation.

The equivocal nature of the influence of variables related to socioeconomic position on public views on forensic DNA testing in the criminal field shows the complexity and dynamic nature of the social representations of what is beneficial and harmful to individuals and society, and how the state-citizen relationship is perceived [55, 60]. Public attitudes towards criminal DNA databases are also embedded in broader cultural and emotional elements that pervade everyday life [58]. The tentative character of public attitudes on forensic DNA testing suggest that other cultural conditioning is to be considered and might correlate with levels of public trust and views about the justice system, drawing attention to the need to include other variables in the analysis of this topic, such as concerns about victimization or excessive police activity [55].

National legislation related to forensic DNA testing vary widely, namely in regard to (a) regulation of forensic DNA databases, in particular criteria for inclusion of DNA profiles and the periods of time and conditions for their retention and/or deletion [6, 9]; (b) regulation of the uses of forensic DNA phenotyping and familial searching [42, 66]; and (c) regimes of data protection and exchange of DNA data across borders [32]. It is necessary to develop an analytical tool that could serve as a basis for comparative cross-national studies covering different regulatory, legal, and political contexts. It would be relevant to develop policies that engage citizens’ perspectives and encourage the participation of scientific actors in the development of anticipatory governance deliberations concerning the widening application of forensic genetics in an increasing number of criminal and civil jurisdictions [12, 23].

Methodological limitations were observed in the existing quantitative research on public views on forensic DNA testing in the criminal field, which is often limited to particular national settings and mainly used nonprobability sampling techniques, with different periods of data collection. There was considerable heterogeneity regarding the topics explored and the use of different categories of analysis to assess exposure to law enforcement occupations. More empirical studies are needed to test the generalization of already known tendencies in different countries.

The results expressed in this scoping review are particularly relevant in a context where the expansion of uses of forensic DNA testing is predictable, in particular in areas which have not yet been regulated (for instance, genetic databases held by commercial companies) [44]. Forensic DNA testing is the commercialization of forensic services requiring more scrutiny since (a) forensic service provision should be for public interest rather than commercial profit [66, 72] and (b) there are new accountability and transparency needs if wider sources of forensic DNA testing lie in the hands of private corporations [44, 72].

## Conclusion

This scoping review raises awareness of the need to expand studies on public views about the role of highly advanced technology in crime fighting. Further quantitative evidence and in-depth qualitative data are required to document collective views. These views on forensic genetic technologies are entangled with assertions about social order, affirmations of shared values and civil rights, and promises about security and justice. In particular, studies about public opinion regarding uses of controversial techniques such as forensic DNA phenotyping and familial searches remain very scarce. Three particular critical aspects are, first, the lack of publicly available information about effective uses of forensic DNA testing in criminal cases, and the corresponding impact in the delivery of justice. Second, although some criminal cases involve the use of familial searching and forensic DNA phenotyping, the use of these technologies remains unregulated in most jurisdictions. Third, there is a lack of public—and policy—discussions regarding whether police should be permitted to access data held by personal genetic service providers, including but not limited to searching genetic genealogy databases for the purpose of generating investigative leads. In this expanding scenario of DNA data stored and used out of centralized criminal DNA databases, there is a deficit of transparency and accountability that requires public discussion on ethically sustainable modes of governance.
